# How to decide intervention thresholds based on FRAX in central south Chinese postmenopausal women

**DOI:** 10.1007/s12020-013-0076-y

**Published:** 2013-10-22

**Authors:** Zhimin Zhang, Yangna Ou, Zhifeng Sheng, Eryuan Liao

**Affiliations:** 1 Institute of Metabolism and Endocrinology, The Second Xiang-Ya Hospital, Central South University, 139 Renmin-Zhong Rd, Changsha, 410011 Hunan People’s Republic of China; 2 Department of Blood Transfusion, The Xiang-Ya Hospital, Central South University, Changsha, People’s Republic of China; 3 Hospital Infection Control Center, The Second Xiang-Ya Hospital, Central South University, Changsha, People’s Republic of China

**Keywords:** Osteoporosis, Intervention thresholds, Fracture risk, FRAX model

## Abstract

The FRAX tool has been used to determine possible thresholds for therapeutic intervention; however, there are no FRAX-based intervention thresholds available for China, we proposed that the 10-year probability of major osteoporotic fracture and hip fracture of about 4.0 and 1.3 %, respectively, may be acceptable intervention thresholds for central south Chinese postmenopausal women.

## Introduction

In recent years, the World Health Organization (WHO) has developed a fracture risk assessment tool termed FRAX. Using easily obtainable clinical risk factors, with or without femoral neck bone mineral density (BMD), FRAX provides models for assessing the 10-year probability of a major osteoporotic fracture and a hip fracture, and determines possible thresholds for therapeutic intervention. However, the WHO makes no specific recommendation concerning intervention thresholds, because these depend on many local factors [1]. WHO suggests that each country should determine their own intervention thresholds, based on the local healthcare situation and the cost-effectiveness of the treatment of osteoporosis.

Clinical guidelines on when to intervene, based on fracture probability, have been developed for Europe, Canada, Germany, Japan, the UK, and the US [2–7]. Two approaches have been developed for guidelines based on fracture probability [8]. The first is to “translate” current practice in the light of FRAX; the UK guidance for the management of men and women at high fracture risk developed by the National Osteoporosis Guideline Group (NOGG) is an example of the translation of existing guidance provided by the Royal College of Physicians (RCP) [9–11] into probability-based assessment [2]. The RCP guidance indicates that treatment should be recommended for women with a prior fragility fracture, without the need for measuring BMD, an approach that has been shown to be cost-effective in women aged >50 years [12]. For this reason, the intervention threshold set by NOGG was at the fracture probability equivalent of women with a prior fragility fracture without BMD testing [2]. The second approach is the determination of the threshold of fracture probability at which intervention becomes cost-effective. The preferred method is cost–utility analysis, which integrates the number of deaths and disability avoided with the multiple outcomes by measuring quality-adjusted life-years (QALYs) [8]. The health-economic thresholds of £30,000 and £20,000 per QALY gained in the UK [13–15], or $60,000 per QALY gained in the US [16], have been used to determine cost-effectiveness. For example, the National Osteoporosis Foundation updated its pre-existing clinical practice guidelines [17] with a health-economic analysis [16]. The threshold for cost-effectiveness was set at $60,000 per QALY gained in both men and women; treatment became cost-effective at a hip fracture probability of approximately 3 %, which was chosen as the intervention threshold.

**Fig. 1:**
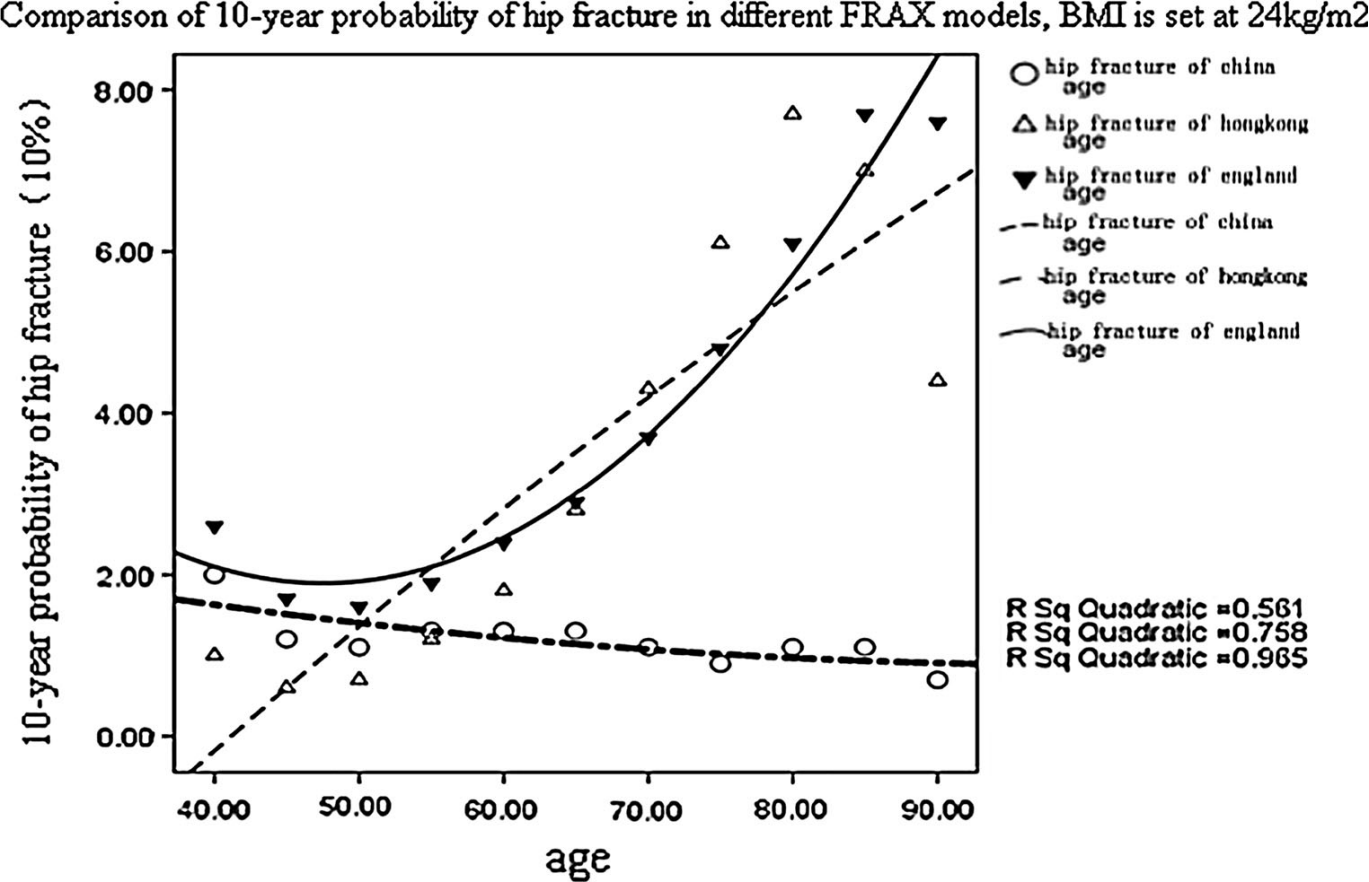
Comparison of 10-year probability of hip fracture in different FRAX models, BMI is set at 24 kg/m^2^

At present, although fracture probabilities derived from FRAX can be computed in some Asian countries; however, there are no specific FRAX-based intervention thresholds available in China. Hong Kong is developing the translational approach to guidelines [14], and the selection of individuals at high risk for treatment in Sri Lankan postmenopausal women is being carried out according to the US Caucasian tool [18]. In Japan, Fujiwara [4] proposed that a 10-year probability of 10 % for osteoporosis-related fracture might be an acceptable intervention threshold. However, it is not suitable to use other countries’ thresholds as a surrogate threshold for China. In a prospective study, the predicted 10-year risk of osteoporotic fracture in Hong Kong Southern Chinese women was substantially higher than that for mainland Chinese women [19]. Kwok [20] found that the prevalence of vertebral fracture was higher in Japan compared with Hong Kong, Thailand, and Indonesia. When the 10-year risk of hip fracture in women with a BMI of 24 kg/m^2^ aged 40, 50, 60, 70, 80, and 90 years with no other clinical risk factors was estimated, the predicted fracture probability in Chinese mainland women was much lower than that in the UK or Chinese Hong Kong, as shown in Fig 1. Moreover, as there is little information on the epidemiology of fracture and death, and systematic drug economics research in China, we cannot determine the intervention thresholds according to the above approaches. For this reason, we want to discuss the interventional thresholds based on FRAX in central Southern China.

We attempted to set intervention thresholds based on the 10-year probability of major osteoporotic fracture and hip fracture calculated with BMD. In our previous study of 778 urban postmenopausal women [21], there were 292 (37.5 %) women with osteoporosis; the osteoporotic women were aged from 51 to 71 years. The 62.5th percentiles of major osteoporotic fracture and hip fracture probability calculated with BMD for the 778 subjects were 4.0 and 1.3 %, respectively. Correspondingly there were 37.5 % subjects who had a 10-year probability of major osteoporotic fracture and hip fracture that exceeded 4.0 and 1.3 %, respectively, and this percentage corresponded to the proportion of subjects with osteoporosis. As FRAX can be used without BMD values, it will provide an interim solution to the limitation in the central-type DXA facility currently observed in China. Thus, we propose that, intervention for both BMD testing and treatment would be recommended for individuals with a 10-year probability of major osteoporotic fracture that exceeded 4.0 % or a 10-year probability of hip fracture that exceeded 1.3 %. However, clinical judgment needs to be used, because the cost-effectiveness of therapeutic intervention could vary depending on the specific drug used, and may avoid costly and potentially unnecessary treatment. In summary, we present a hypothesis that the 10-year probabilities of major osteoporotic fracture and hip fracture of about 4.0 and 1.3 %, respectively, may be currently acceptable as the intervention thresholds for central Southern Chinese postmenopausal women; however, the decision to intervene should take account of health and economic consequences for individuals and for the health care budget. It is noteworthy that China is a large country with a heterogeneous population, while only a small sample size was included in our analysis; therefore, our conclusion will require further confirmation. However, it may serve to suggest FRAX-based intervention thresholds in the Chinese setting. The adoption of FRAX-based intervention thresholds will demand a reappraisal of the criteria for reimbursement of interventions and health-economic assessments.
